# A prospective study of the effect of delivery type on neonatal weight gain pattern in exclusively breastfed neonates born in Shiraz, Iran

**DOI:** 10.1186/1746-4358-5-1

**Published:** 2010-01-27

**Authors:** Azadeh Saki, Mohammad R Eshraghian, Kazem Mohammad, Abbas Rahimi Foroushani, Mohammad R Bordbar

**Affiliations:** 1 Department of Biostatistics, School of Public Health and Institute of Public Health Research, Tehran University/Medical Sciences, Tehran, Iran; 2 Department of Pediatrics, School of Medicine, Shiraz University of Medical Sciences, Shiraz, Iran

## Abstract

**Background:**

In this exploratory study, the contribution of delivery type to the weight gain pattern for full-term infants with exclusive breastfeeding in the first month of infancy was determined. In addition, breastfeeding success among cesarean section (C-section) delivery mothers based on their neonate's weight gain at the end of the first month of infancy was evaluated.

**Methods:**

A cohort of 92 neonates born in Shiraz, from July 10 to August 10, 2007 was followed longitudinally. The data were collected during the first month postpartum at three occasions: 3 to 7 days postpartum, 10-21 days postpartum and 24-31 days postpartum.

**Results:**

Among 92 mothers in this study, 35 (38%) were delivered by C-section. Generalized estimating equation (GEE) showed that delivery type (p < 0.01), receipt of advice about breastfeeding (p = 0.03) and neonate's age (p < 0.01) significantly affected weight gain. GEE estimated the values of the parameters under study and the testing contribution of each factor to weight gain, leading to the conclusion that gender, parities and maternal education did not contribute to weight gain. The neonate's weight gain pattern for C-section deliveries lies below that of normal vaginal deliveries until 25 days postpartum, when weight gain for C-section deliveries became higher than that for normal vaginal deliveries.

**Conclusions:**

Type of delivery contributes strongly to the weight gain pattern in the first month of infancy. In spite of greater weight loss among C-section birth neonates in the first days of life, at the end of the first month neonates showed a similar weight gain. Consequently, mothers with C-section delivery can successfully exclusively breastfeed.

## Background

It is normal for newborns to lose weight during the first days of life. Although much of this weight loss is thought to be due to changes in the volume and distribution of water in the body, some studies show that early skin-to-skin contact, initiating breastfeeding as soon as possible, and feeding practices also influence the degree of weight loss [1-5]. Dehydration and/or failure to thrive during the first days postpartum may occur as a result of lactation failure and lack of awareness about feeding problems. Recent reports recommend monitoring infants' weight through the neonatal period [6-8].

Extensive research on the biology of human milk and health outcomes associated with normal methods of infant feeding have established that breastfeeding is more beneficial than formula feeding, although breastfed infants initially lose more weight and take longer to regain their birth weight than formula fed infants [9-14]. One of the factors affecting breastfeeding initiation and duration is birth by cesarean section [15-17].

In recent years C-section has been performed upon request for births that would otherwise have been vaginal [18,19]. In Iran, the C-section rate is about 50%-65%, however in some private hospitals the rate reaches 90% [20].

A study in Mexico shows that C-section is a risk factor for not initiating breastfeeding (Odds Ratio, OR = 1.56) and for breastfeeding for less than one month (OR = 1.72), but it is unrelated to the duration of breastfeeding among women who breastfeed their babies for one month or more (OR = 1.03) [15]. Similar studies performed in Puerto Rico and Athens show that C-section delivery was negatively related to breastfeeding initiation (OR = 1.56, OR = 4.16) [16,17]. Breastfeeding in post-cesarean women has a protective effect on infant health, as demonstrated by the decrease in illness-related hospitalizations in the first year of life [21].

International agencies emphasize the need for exclusive breastfeeding during the first months of life. A new international growth standard chart has been prepared based on children who are fed according to World Health Organization recommendations, which entail exclusive breastfeeding for the first six months of life [22]. An increase in weight indicates a child's well-being. The rate of growth is relatively high during an infant's first months of life and is susceptible to decelerating forces that may compromise a child's ultimate level of growth. Research evidence showed both biological and experiential conditions influence growth. Biologic conditions that may influence these components include gender and gestational maturity at birth, that is, premature or full-term. Mother's care-giving and infant feeding behavior also influence infant growth [23,24].

The neonate growth pattern does not have a uniform rate of increase during the first month postpartum. Weight loss, rather than weight gain, may occur in the first week postpartum.

To the best of our knowledge, there are no studies exploring the relationship between the type of delivery (cesarean-section (CS) or normal vaginal (NV)) and the pattern of neonatal weight gain during the first month. Therefore, the purpose of this study is to explore the impact of type of delivery on the pattern of weight gain for exclusively breastfed neonates through the first month postpartum. A second purpose is to compare infant weight at first month postpartum in exclusively breastfed infants who were born by vaginal birth or cesarean-section.

## Methods

### Sample collection and follow-up

A sample of mothers of singleton full-term infants weighing ≥ 2500 g who were exclusively breastfeeding [25] and presented at Shiraz health care centers within three to seven days postpartum were recruited to participate in the study. All the mothers initiated breastfeeding within two hours after birth. The participants were recruited from 10 July to 10 August 2007 and were followed up for one month. Initially, 104 mother-infant pairs were recruited. However, if the mothers reported they were no longer exclusively providing breast milk to their neonate at follow-up visits they were excluded from further participation in the study. In addition, if the neonate was hospitalized during the study, the mother-infant pair was excluded. Based on these criteria, 10 (9.6%) mother-infant pairs [4 (10.3%) from the CS group and 6 (9.5%) from the NV group] were excluded because of formula use and 2 (1.9%) mother-infant pairs (one from the CS group and another from the NV group) were excluded due to infant hospitalization during the study. Thus, at the end of the study, 12 pairs were excluded, leaving 92 (88.5%) mothers and their exclusively breastfed neonates remaining.

Two data collection tools were developed by the researchers; The first questionnaire, completed at the time of recruitment to the study, included maternal and neonatal demographic and background data [neonate's date of birth, gender, birth weight and birth length (according to hospital records); mother's age, education, smoker (yes/no), type of delivery (NV/CS), type of maternity hospital (private/public), parity, previous breastfeeding experience (yes/no), and family income]. The second questionnaire, completed at recruitment and then at the second and third occasions, was used to collect neonatal assessment data (anthropometric measures and health status), current feeding pattern [breastfeeding exclusively ("Have you fed your baby anything except mother's milk?")]. Mothers were also asked if they received professional advice about breastfeeding at healthcare centers or maternity hospitals ("Have you received advice about breastfeeding till now?").

The questionnaires were completed during the first month at three occasions: 3 to 7 days postpartum, 10-21 days postpartum and 24-31 days postpartum, when mothers presented in healthcare centers. One nurse at each of the seven healthcare centers was responsible for completing the neonatal assessment and questionnaires. The nurses were trained in the measurement of anthropometric indices by a healthcare center physician. Inter-rater reliability among the nurses was checked by the inspectors of the Deputy for Health at Shiraz University of Medical Sciences.

The weights were measured to the nearest 10 grams on sophisticated balance scales calibrated at each healthcare center. The nurses were instructed to weigh the neonates naked. If this was not possible, the type of clothing was recorded. Later, using these data, the neonate's weight was adjusted for baby clothes. Age at each measurement was recorded exactly based on the difference between the date of measurement and date of birth in days.

### Statistical analysis

The sample size for each group of delivery types required for detection of a meaningful difference in the neonatal weight gain, Δ, with a desired power 1-β at α level of significance is given by:

Where σ*^2 ^= 29.16 is the sum of the variance components obtained with a pilot study (n = 10). The power of the study was 80% with α = 0.05 and the meaningful difference between CS and NV birth neonates considered as 5 g. Assuming 20% withdrawal, n ≥ 37 for each group were required. Therefore we continued data collection until we had 40 CS delivery mothers; in this period 64 NV delivery mothers were recruited.

Generalized Estimating Equation (GEE) modeling was used to determine factors related to neonatal weight gain. The Generalized Estimating Equation approach is widely used in biomedical sciences for the analysis of longitudinal data. Models such as Generalized Estimating Equation allow for the correlation structure in the data due to the repeated measurements on the same subjects over time. Generalized Estimating Equation modeling has many attractive robust properties and consistent parameter estimations that are not prejudiced by incorrect specification of the correlation structure. The GEE approach is based on the concept of "estimating equations" and provides a very general and unified approach for analyzing correlated responses that can be discrete or continuous. The essential idea behind the GEE approach is to generalize and extend the usual likelihood equations for a generalized linear model for a univariate response by incorporating the covariance matrix of the vector of responses [26-32].

Locally Weighted Scatterplot Smoothing (LOESS) approach was also used to obtain weight gain pattern. The LOESS is one of many "modern" modeling methods that build on "classical" methods, such as linear and nonlinear least squares regression. LOESS allows greater flexibility because no assumptions about the parametric form of the regression surface are needed. Response y_i _and corresponding predictor measurement x_i _were related by y_i _= g(x_i_) + ε_i_, for i = 1,..., n, where g is the regression function. A local approximation is obtained by fitting a regression surface to the data points within a chosen neighborhood of the point x [33].

Independent T-test was used to compare the mean of different factors between CS and NV delivery type. Chi-square test was also used to investigate the association between family income and maternal education level with types of delivery. P-values less than or equal to 0.05 were considered significant. SPSS11.5 and S-Plus2000 statistical software were used for data analysis.

### Ethical considerations

Ethical approval was obtained from the Ethic in Research Committee at the Deputy of Research of Tehran University of Medical Sciences. There were no anticipated physical, social or legal risks associated with participation. Informed consent was implied if participants completed the first questionnaire. It is standard practice in Iranian healthcare centers to ask participants to complete questionnaires at health checks without written consent.

## Results

At the end of this study we had 92 mother/infant pairs. Among them, 57 (62%) mothers had NV delivery and 35 (38%) had CS delivery. Table 1 presents descriptive statistics for all background variables from mothers and infants by type of delivery. Among the mothers with CS delivery, 20 (57%) were primiparous and among the mothers with NV delivery, 32 (56%) were primiparous. All forty multiparous mothers had previous breastfeeding experience. None of the mothers were cigarette smokers. There was no significant difference in maternal education, mother's age, parity, neonate's gender, birth weight and birth length between CS and NV deliveries (p > 0.05). Chi-Square testing showed that family income was a very strongly related factor with the method of delivery (p < 0.01). Eighty percent of mothers who lived in excellent income families had CS delivery. This percentage was, respectively, 44%, 31% and 0% for good, fair and poor income families. Also, the rate of CS was significantly different (p < 0.01) between private and public hospitals. Most CS delivery mothers (77%) received advice about breastfeeding at the first occasion, but among NV delivery mothers this rate was 47%. The proportion of mothers who received advice about breastfeeding increased during the study, with significantly higher proportions among the CS delivery mothers at all occasions (p < 0.05).

**Table 1:** Descriptive statistics for background variables for mother and infant by type of delivery

Variables	Normal vaginal delivery (n = 57)	Cesarean section (n = 35)	Total (n = 92)	p-value
Neonate's gender				0.11*
Girl, n (%)	30 (53)	13 (37)	43 (47)	
Boy, n (%)	27 (47)	22 (63)	49 (43)	

Birth weight (g), mean (SD)	3130.3 (432.1)	3145.1(450.9)	3136.0(436.9)	0.87**

Birth length (cm), mean (SD)	50.0 (1.9)	50.0 (2.7)	50.0(2.2)	0.99**

Mother's age, mean (SD)	23.6 (4.4)	23.9 (3.8)	23.7(4.1)	0.71**

Maternal education				0.60*
Primary, n (%)	7 (12)	4 (11)	11 (12)	
Secondary, n (%)	20 (35)	9 (26)	29 (31)	
University, n (%)	30(53)	22 (63)	52 (56)	

Maternal smoking, n (%)	0	0	0	1.00*

Parity				0.55*
Primipara, n (%)	32 (56)	20 (57)	52 (56)	
Multipara, n (%)	25 (44)	15 (43)	40 (44)	

Family income				<0.01*
Poor, n (%)	6 (11)	0 (0)	6 (7)	
Fair, n (%)	27 (47)	12 (34)	39 (42)	
Good, n (%)	13 (23)	10 (29)	23 (25)	
Excellent, n (%)	2 (3)	8 (23)	10 (11)	
Missing, n	9 (16)	5 (14)	14 (15)	

Birth hospital				<0.01*
Private, n (%)	4 (7)	15 (43)	19 (36)	
Public, n (%)	53 (93)	20 (57)	73 (64)	

Received advice about breastfeeding				
First occasion, n (%)	27 (47)	27 (77)	54 (59)	0.01*
Second occasion, n (%)	43 (75)	32 (91)	75 (82)	0.04*
Third occasion, n (%)	45 (79)	33 (94)	78 (85)	0.04*

* p-value calculated with Chi-Square test.

** p-value calculated with t-test.

**Figure 1:**
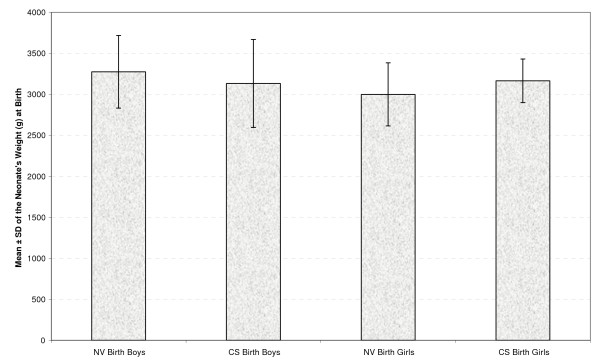
**Mean ± standard deviation (SD) of the neonate's weight (g) by gender and delivery type at birth**.

**Figure 2:**
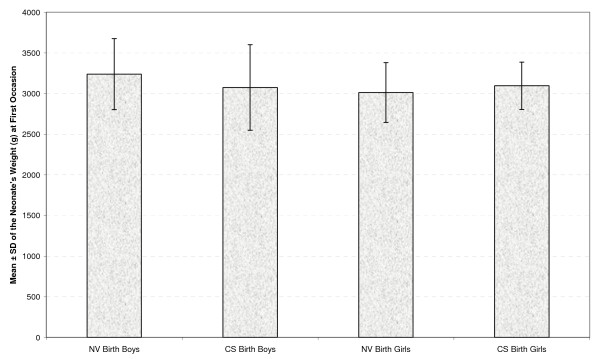
**Mean ± standard deviation (SD) of the neonate's weight (g) by gender and delivery type at first occasion**.

**Figure 3:**
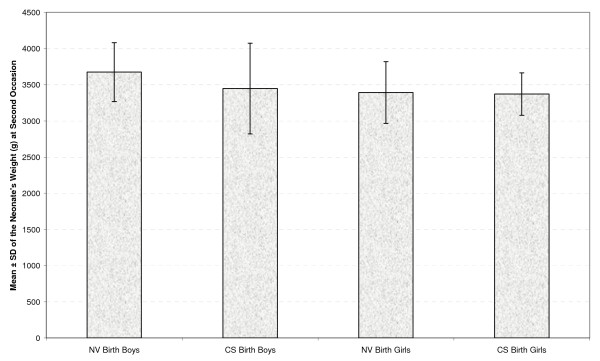
**Mean ± standard deviation (SD) of the neonate's weight (g) by gender and delivery type at second occasion**.

**Figure 4:**
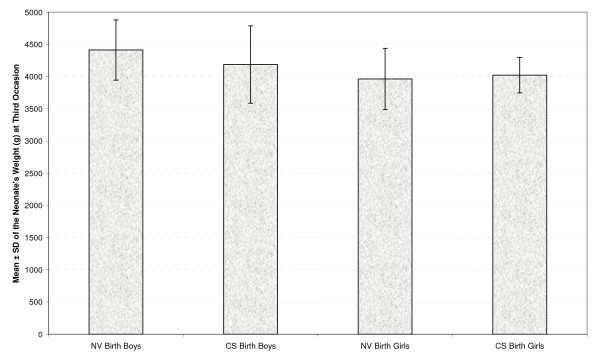
**Mean ± standard deviation (SD) of the neonate's weight (g) by gender and delivery type at third occasion**.

Table 2 presents the mean and standard deviation of the neonates' weight at four occasions by gender and type of delivery (Figures 1, 2, 3 &4). As seen in all previous studies, the boys' weight is greater than that of the girls in all occasions and for both types of delivery.

**Table 2:** Mean and SD of the neonate's weight (g) at different occasions by gender and delivery types

Gender	Type of delivery	At birth		First occasion (median 5 days)		Second occasion (median 15 days)		Third occasion (median 30 days)	
		
		Mean	SD	Mean	SD	Mean	SD	Mean	SD
Boy	NVD	3275.2	442.4	3238.9	436.4	3675.9	407.0	4413.6	467.3
	CS	3133.2	537.0	3075.0	527.1	3447.6	627.6	4187.5	600.8

Girl	NVD	3000.0	384.6	3013.3	368.1	3393.3	427.0	3963.5	474.9
	CS	3165.4	265.7	3096.2	291.9	3371.5	292.6	4023.1	275.8

In addition to the type of delivery, some factors such as gender, mother's education, received advice about breastfeeding (mother's knowledge about breastfeeding) and parity (mother's experience) were included in the model. Table 3 presents the results of model fitting and parameter estimates as well as the results of testing the contribution of each factor to weight gain according to generalized estimating equation model. This demonstrates that, except for the type of delivery (p = 0.01), receiving advice about breastfeeding (p = 0.03), age (p < 0.01) and interaction between age and type of delivery (p = 0.02), the other factors do not contribute significantly to weight gain at different times during the first month of infancy. Therefore, they were excluded from the model.

**Table 3:** Parameters estimation, standard errors and p-values from fitted model for neonatal weight gain at three occasions

Variable	Parameter estimate	Standard error	p-value
Type of delivery	14.4	5.4	0.01
Received advice about breastfeeding	7.7	3.5	0.03
Type of delivery * neonate age	-0.5	0.2	0.02
Neonate age (in days)	2.0	.2	< 0.01

**Figure 5:**
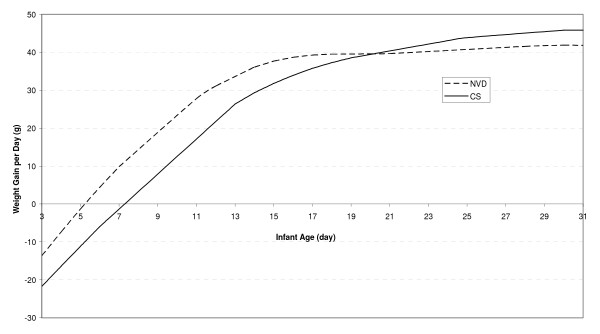
**Smoothed weight gain versus age curve for neonates by delivery type**.

Figure 5 presents a smooth weight gain curve for neonates aged 3-31 days old separately for NV and CS deliveries. It can be seen from Figure 5 that weight gain values for the first 5 days of NV deliveries and the first 7 days of CS deliveries of postpartum are negative, which means weight loss for both groups in this period. Neonates with CS deliveries lost more weight and took longer to regain their birth weight than NV deliveries. Also, it can be seen that the weight gain pattern for CS deliveries is lower than that of NV deliveries until 25 days postpartum. At this time the pattern starts to rise and continues until 31 days postpartum.

**Figure 6:**
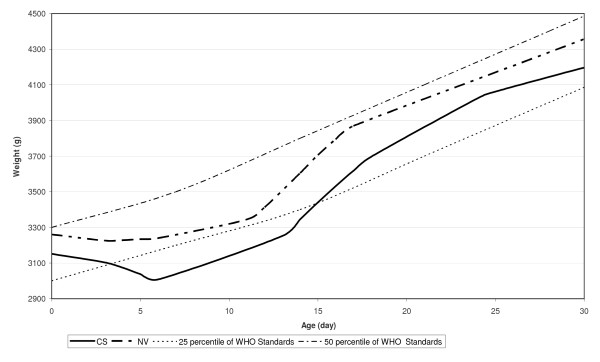
**Smoothed weight versus neonate's age curves for boys by delivery types compared with fifty percentile of WHO Child Growth Standards**.

**Figure 7:**
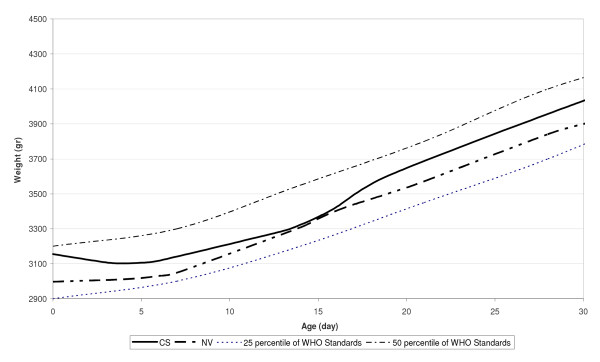
**Smoothed weight versus neonate's age curves for girls by delivery types compared with fifty percentile of WHO Child Growth Standards**.

In Figures 6 and 7 our fitted curves for neonatal weight were compared with the 25th and 50th percentiles of the WHO growth standard charts, for boys and girls respectively. Figure 6 shows that the mean weight of boys with CS delivery at birth is below the 25 WHO growth standards percentile, but at the end of the first month, this reached a higher level than the 25 WHO growth standards percentile. Also, the mean weight of boys with NV delivery at birth is a little higher than the 25 WHO growth standards percentile, but at the end of the first month, this reached about 50 percentile. Figure 7 shows that the mean weight of girls with CS delivery at birth is higher than the 25 WHO growth standards percentile, but at the end of first month, this approached 50 percentile. Also, the mean weight of girls with NV delivery at birth is less than the 25 WHO growth standards percentile, but at the end of the first month this reached higher than 25 percentile.

## Discussion

The most important finding of this study was the strongly significant association between type of delivery and neonatal weight gain. According to the results displayed in Table 3, neonates born by vaginal birth gained 14 gram weight per day more than those born by cesarean section. In Iranian hospitals, most of the C-sections are performed with general anesthesia, so very early skin-to-skin contact and breastfeeding occur immediately post-cesarean. That negatively affects milk supply and breastfeeding practices, and as a result neonates' weight gain during the early postpartum period. However, due to the significant negative interaction between the infant's age and type of delivery, this difference has decreased over time. This means that the effect of CS delivery has been reduced as well. Therefore, as seen in Figure 5, the CS weight gain curve reaches the NV weight gain curve in 25 days postpartum. After 25 days, the weight gain for CS deliveries is significantly greater than that in NV deliveries. We found that infant gender, maternal education and parity did not contribute significantly to weight gain during the first month of infancy.

Receiving advice about breastfeeding is another significant factor for neonatal weight gain. As shown in Table 3, neonates whose mothers received advice about breastfeeding gain 7.7-gram weight more than those who did not. This factor does not interact with age, but does have a constant effect during the first month of infancy. Higher rates of receiving advice about breastfeeding among CS delivery mothers (Table 1) may have helped them in successful breastfeeding practices and improve their neonate's weight gain at the end of the first month following CS.

No previous growth charts, including World Health Organization Growth Standards Charts, have focused on the first month of infancy [34]. A monotonous increase in weight is shown, but most of the breastfed neonates lose weight in the first days postpartum. As shown in Figure 5, neonatal weight decreased in the first days of life (weight loss) and after 5-7 days increased (weight gain). Therefore, we obtained a weight chart for the first month of infancy by age and delivery type separately for boys (Figure 6) and girls (Figure 7). These figures show that, disregarding the type of delivery, weight of the neonates that exclusively breastfed at the end of the first month was higher than their standard percentiles at birth.

For some mothers, the reason for the neonate's weight loss in the first days postpartum is their insufficient milk. This causes them to use formula feeding and may lead to early breastfeeding cessation [35]. The result of this study shows that (Figure 5) the weight gain pattern improves after the first week and mothers can be hopeful that their neonates will gain more than 40 grams of weight per day.

The model used to investigate the related factors in this research is more robust than those examined in earlier studies. The advantage of these models compared with other methods in this area is that the size of each covariate effect using regression model parameters is introduced.

## Conclusion

Gender, mother's education and parity did not contribute to weight loss in the first days postpartum; however delivery type and receiving advice about breastfeeding contribute strongly to the weight gain pattern in the first month of infancy. Neonates with CS delivery in the first days postpartum lose more weight than those with NV delivery; however at the end of the first month there is no difference between the weights of breastfed infants born by CS or NV delivery. Consequently, if mothers with CS delivery continued exclusively breastfeeding they could have successful breastfeeding and these results lead to calling for early skin-to-skin contact and support of professionals and family post-cesarean delivery.

## Competing interests

The authors declare that they have no competing interests.

## Authors' contributions

AS was the principal investigator, collected and analyzed the data and drafted the manuscript. MRE conceptualized and supervised the study, reviewed the analyses and also the final discussion. KM and ARF acted as consultants and reviewed the manuscript. MRB reviewed the results and helped to improve the discussion. All authors have read and approved the final manuscript.
